# Subtype classification and functional annotation of L1Md retrotransposon promoters

**DOI:** 10.1186/s13100-019-0156-5

**Published:** 2019-04-08

**Authors:** Meng Zhou, Andrew D. Smith

**Affiliations:** Molecular and Computational Biology Section, Division of Biological Sciences, University of Southern California, Los Angeles, USA

**Keywords:** Retrotransposon, L1Md monomer, Classification, Annotation

## Abstract

**Background:**

L1Md retrotransposons are the most abundant and active transposable elements in the mouse genome. The promoters of many L1Md retrotransposons are composed of tandem repeats called monomers. The number of monomers varies between retrotransposon copies, thus making it difficult to annotate L1Md promoters. Duplication of monomers contributes to the maintenance of L1Md promoters during truncation-prone retrotranspositions, but the associated mechanism remains unclear. Since the current classification of monomers is based on limited data, a comprehensive monomer annotation is needed for supporting functional studies of L1Md promoters genome-wide.

**Results:**

We developed a pipeline for *de novo* monomer detection and classification. Identified monomers are further classified into subtypes based on their sequence profiles. We applied this pipeline to genome assemblies of various rodent species. A major monomer subtype of the lab mouse was also found in other *Mus* species, implying that such subtype has emerged in the common ancestor of involved species. We also characterized the positioning pattern of monomer subtypes within individual promoters. Our analyses indicate that the subtype composition of an L1Md promoter can be used to infer its transcriptional activity during male germ cell development.

**Conclusions:**

We identified subtypes for all monomer types using comprehensive data, greatly expanding the spectrum of monomer variants. The analysis of monomer subtype positioning provides evidence supporting both previously proposed models of L1Md promoter expansion. The transcription silencing of L1Md promoters differs between promoter types, which supports a model involving distinct suppressive pathways rather than a universal mechanism for retrotransposon repression in gametogenesis.

**Electronic supplementary material:**

The online version of this article (10.1186/s13100-019-0156-5) contains supplementary material, which is available to authorized users.

## Background

Long interspersed nuclear element-1 (LINE-1 or L1) is a major type of autonomous retrotransposons that is commonly found in all mammalian genomes [1]. L1 elements act as parasitic retro-elements in the host genome via a copy-and-paste mechanism to increase their copy numbers in the genome [2, 3]. They comprise about 17% of the human genome and 19% of the mouse genome [4, 5]. A typical full-length L1 element is about 6–7 kb long, containing a 5’ untranslated region (UTR), two open reading frames (ORF1 and ORF2) and a 3’ UTR [6–9]. The 5’ UTR harbors transcription factor binding sites and serves as an internal promoter for transcription by RNA polymerase II [10]. Families of L1 found specifically in the mouse genome are named after the species name of the lab mouse *Mus domesticus*: the L1Md families [11]. A special tandem repeat structure has been found in L1Md 5’ UTRs, with each repeating sequence called a monomer [12, 13]. So far such tandem repeat structure has also been observed in other genomes, but only those in the mouse genome are located close to the 5’-end of L1 elements, and the number of monomers per retrotransposn is much higher in mouse [14]. The tandem repeat structure is thus a special feature of L1Md promoters, and it makes studying the transcription activity and regulation of L1Md elements a challenging task.

To generate inheritable insertions, retrotransposons must propagate in the genome during germ cell development or early embryogenesis. This also means the host must prevent retrotransposon proliferation at the same time to effectively protect genome integrity at specific loci and preserve overall genome size. DNA methylation is an important epigenetic mark that is employed to regulate retrotransposon expression in vertebrates and plants [15–17]. While DNA methylation is being constantly maintained in most cell types, it is erased and re-established during the epigenetic reprogramming of germ cell development in almost the entire genome [18]. This process potentially leaves a window for retrotransposon transcription. It has been shown that by default most of the genome is quickly re-methylated during reprogramming, except for regions that are occupied by active chromatin marks [19], likely indicating occupancy by some DNA-binding protein directly interfering with the addition of methyl groups. Nevertheless, this does not guarantee repression of already actively expressed retrotransposons. It is known that proteins of the Piwi family are responsible for regulating retrotransposon expression levels through a conserved Piwi-interacting RNA (piRNA) pathway, which specifically targets retrotransposon transcripts in germ cells [20, 21]. The piRNA pathway is also found to guide *de novo* DNA methylation to silence retrotransposon promoters [22, 23]. Since this mechanism is highly sequence-sensitive, a detailed classification of retrotransposons, especially their promoters, can provide insight into the regulation of retrotransposon transcription.

The monomer structure increases the difficulty of both the classification and functional annotation for L1Md elements. It remains elusive how L1Md elements adopted the tandemly repeating monomers as their promoters during evolution. Since it was shown that multiple monomers in one promoter have linearly additive effect for transcription activity [24, 25], it is possible that the promoter expansion mechanism has also elevated the transcription activity of L1Md elements. However, a genome-wide annotation of L1Md promoter activity is still lacking.

Due to the retrotransposition mechanism, most L1Md elements are 5’-truncated [11], resulting in retro-elements incapable of initiating transcription. Thus, although the total number of L1 elements in the mouse genome is in the order of hundreds of thousands, the number of promoter-containing full-length L1 elements is estimated to be less than 20 thousand [26, 27]. The state-of-art classification of mouse L1 elements includes 29 families [26]. In most cases, L1Md families are named after their promoter types, which are determined by the type of monomers contained in one promoter. Currently there are three known monomer types: A, G_f_ and T_f_ [12, 28, 29]. The latter two originated from one common ancestor type, the F type [13], and there is no significant sequence similarity between the A type and other monomer types. These three monomer types are active in terms of transcription, and therefore L1Md families which contain these monomers are likely to be capable of retrotransposition. In addition to the monomer type classification, it has been shown that the A type can be further divided to six subtypes based on sequence difference at a finer scale [30]. However, this subtype definition is based on a limited number of sequences, and it is unclear whether any unknown A subtypes exist in the mouse genome. To the best of our knowledge, no subtype identification of the G_f_ and T_f_ types has been reported.

Annotation of L1 elements in practice usually emphasizes the termini of consensus sequences. For example, in the most commonly used database of retrotransposon consensus sequences, RepBase [31], sequences related to L1 are stored as the 5’-end, including 5’ UTR and ORF1, and the 3’-end, including ORF2 and 3’ UTR. The names of these consensus sequences are used by the annotation software RepeatMasker [32] for family classification of retrotransposons. Given the fact that recombination between retrotransposons frequently occurs during evolution [26, 33], it is possible that the ORF1 and promoter of a single L1 element can actually belong to different families, and thus the resulting classification can be ambiguous and misleading when using the annotation information for a retrotransposon as the basis for its biological inference. For example, an L1 element may have switched its promoter from F type to A type due to some rearrangement event. This could alter transcriptional behavior, but such a retrotransposon would remain classified as an L1MdF element based on downstream sequences. In a comprehensive analysis of transcriptionally active retro-elements, such retrotransposons would be excluded because of their classification.

Here we present a *de novo* monomer detection pipeline to precisely locate tandemly repeated sequence in the 5’ UTR of L1 elements. This pipeline uses profile-HMM to improve the sensitivity and accuracy of monomer detection. After detection, monomers are comprehensively considered for identification of subtypes. We show that the current subtype classification of Type A monomers can be greatly expanded, and the subtypes of G_f_ and T_f_ type monomers are also discovered. Our stochastic model based definition of subtypes can be used to represent the diverse with-in group variance of monomers, which reflects their evolutionary ages. Based on this framework, we annotate the activity of L1Md promoters during germ cell development using epigenetic data. These results suggests that the regulation of piRNA-guided *de novo* DNA methylation on functional L1Md promoters is through pairings of specific monomer subtypes and individual activities that comprise the piRNA pathway.

## Results

### Pipeline for monomer detection

**Fig. 1:**
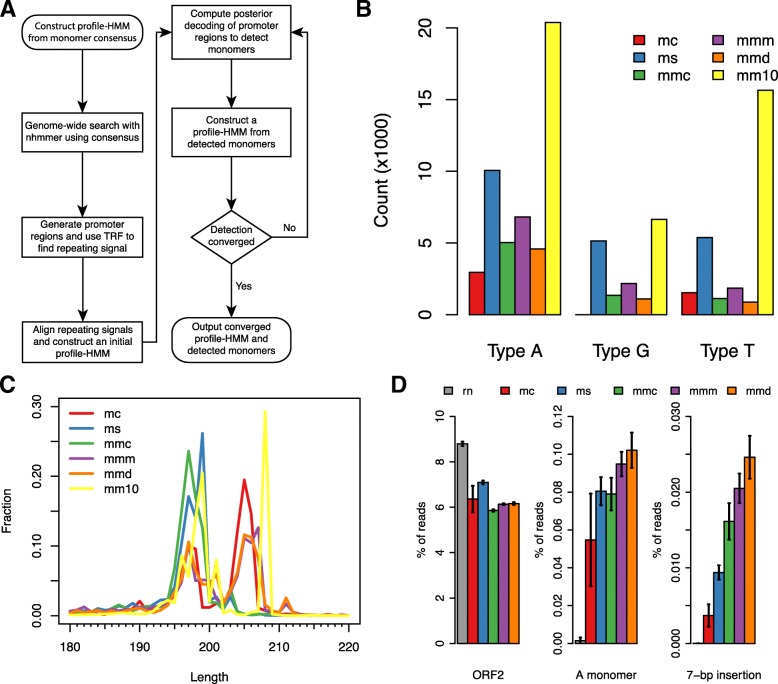
Application of monomer detection pipeline on various mouse genome assemblies reveals potential species-specific monomer subtypes. In this figure, genome names are abbreviated using the initials of their corresponding species name, except for mm10. They are: rn (*Rattus norvegicus*), mc (*M. caroli*), ms (*M. spretus*), mmc (*M. m. castaneus*), mmm (*M. m. musculus*), and mmd (*M. m. domesticus*). **a** Workflow of the monomer detection starting from a single consensus sequence. **b** Count of identified monomers in six mouse genomes. For each monomer type in all genomes, its corresponding consensus sequence reported in previous literature was used as the starting heuristic. **c** Length distribution of detected Type A monomers within the range of 180 – 220 bp. **d** Percentage of reads detected to share sequence similarity with L1 ORF2, Type A monomer and the A monomer subtype with a specific 7-bp insertion, respectively. Error bars indicate ±SD

In L1Md promoters, monomers are organized in tandem, with consecutive monomers being located immediately adjacent to each other [30]. However, we found that commonly used sequence homology searching tools, for example nhmmer [34] and blat [35], tend to leave small gaps (2 to 3 bp) between detected monomers (data not shown). Such small gaps would have confounding effects for solving the exact boundaries of individual monomers, because consecutive monomers form periodic sequences, and gaps would make it hard to precisely delimit individual monomers. Thus we developed a pipeline based on profile-HMM to accurately detect individual monomers. The topology of this model is analogous to that introduced by Durbin, et al. [36], with specific modifications to fit the tandem array of monomers (Additional file 2: Figure S1). The workflow of this pipeline is shown in Fig. 1a. In this pipeline, a homology searching tool is first used to locate potential L1Md promoters (we employ nhmmer for this), where TandemRepeatsFinder (TRF) [37] is applied to detect the repeating sequences. These tandem sequences are used to construct an initial profile-HMM through multiple sequence alignment. Then the initial profile-HMM is refined by iteratively scanning the promoters and estimating emission parameters based on sampling of top-scoring sequences. This process will terminate when the detected boundaries become stable between iterations (see Methods).

We applied our pipeline separately for detection of the three known monomer types: A, G_f_ and T_f_ in the *Mus musculus domesticus* C57BL/6J genome (version mm10), and additional mouse genome assemblies released by the Mouse Genome Project [38], including CAROLI/EiJ (*M. caroli*), SPRET/EiJ (*M. spretus*), CAST/EiJ (*M. m. castaneus*), PWK/PhJ (*M. m. musculus*), and WSB/EiJ (*M. m. domesticus*). The Type F monomer was not included because it is the common ancestor of Type G_f_ and T_f_, which would create overlap in the nhmmer detection results. Hereafter we abbreviate G_f_ and T_f_ as Type G and T, respectively. The number of detected monomers in each genome is shown in Fig. 1b. We found that the number of monomers was significantly lower in all other genomes compared to mm10, even for the WSB/EiJ strain, possibly due to difficulty in assembly construction for highly similar monomers. We examined the amount of unassembled nucleotides proximal to detected monomers. Indeed there is a substantial fraction of unassembled regions around detected monomers, but most of them are located to the downstream region (Additional file 2: Figure S2). We speculate that there are more such regions that cover entire L1Md promoters, which could cause monomers in such regions to be undetectable.

The finalized profile-HMMs for all three types of monomers in the mm10 genome are presented as consensus sequences in Additional file 2: Figure S3. In our detection results, the T consensus has a shift of periodicity compared with the previously reported one. This is probably due to the fact that some T promoters have truncated monomers in the 3’-end region [28]. Because currently only Type A monomers have well defined subtypes based on indels [30], we chose to examine the length distribution of Type A monomers detected using our pipeline to investigate if there is any unseen monomer variant. We found that the range for all six A subtypes (197 – 208 bp) covers monomers in all genomes very well, while lengths in the range of 202 bp to 207 bp are more abundant in genomes other than mm10 (Fig. 1c). The most striking difference is that the 208 bp length is almost completely missing in all other genomes except for mm10. According to the previous A subtype definition [30], the 208 bp monomers are classified as the A-I subtype based on a 7-bp insertion. Since A-I is the youngest and most abundant subtype in mm10, it is unlikely that all other species and strains lack this subtype of monomer.

We then verified the existence of A-I in the five mouse species other than lab mouse using raw genomic sequencing reads. The reads were obtained from the same data set released by the Mouse Genome Project [38] for genome assembly construction. In addition, we included two genomic sequencing samples for rat as an outgroup control [39]. We first used nhmmer to search for ORF2-related reads as positive control, and then applied the same procedure to search for Type A monomer related-reads (see Methods for thesholds). For reads that are found to be related with Type A monomer, we used our customized profile-HMM to detect the 7-bp insertion through posterior decoding (see Methods). The percentages of reads that are related to these three categories are shown in Fig. 1d, and the statistics of all samples used for this analysis are included in Additional file 1: Table S8. As expected, there are very few reads detected to be related with Type A monomer in rat, although rat has a higher fraction of ORF2-related reads. It can be seen that the fractions of ORF2-related reads are similar across all *Mus* species, but the fraction differs for Type A monomer and the A-I subtype. A probable cause for this bias is that the sequences used in this analysis are from the lab mouse genome. Therefore the number of reads detected to be related to A monomers and potential A-I subtype is a function of evolutionary distance between the corresponding species to lab mouse. Despite this bias, we believe that this result is still sufficient to support that the A-I subtype monomers which are commonly observed in lab mouse also exist in other *Mus* species, because of the presence of the specific 7-bp insertion. This indicates that this specific subtype existed in the common ancestor of *Mus*, which can be dated back to around 4 million years ago.

### Identification and characterization of monomer subtypes

The classical subtype definition of Type A monomers is problematic for direct application to infer subtypes genome-wide, because it is based on features that are confounded by random indels that arose during evolution. Therefore we developed an unsupervised approach which uses information independent of sequence length to identify and annotate monomer subtypes in the whole genome. The profile-HMM implemented for this study allows the computation of Fisher score vectors [40, 41], which can be used for subtype identification instead of raw DNA sequences. The Fisher score vector given a profile-HMM is a vector of partial derivatives of emission parameters of the log-likelihood of the observed sequence. The key advantage of using this approach is that all sequences can be converted to a vector of the same dimension, which automatically solves the problem of altered lengths caused by random indels.

To mitigate the effect of truncation and long indels, we defined a set of “core” monomers. This set only consists of monomers with length ranging from 180 bp to 220 bp with the additional requirement of having another monomer both 5’ and 3’. The length range was set empirically based on the length distribution of detected monomers. Monomers not meeting these criteria were excluded from subtype identification as they are likely truncated. Because the Y chromosome has not yet been included in genome assemblies released by the Mouse Genome Project [38], we decided to only include L1 elements located on autosomes and the X chromosome to make the analysis comparable across different mouse species. A principal component analysis (PCA) was first applied on the Fisher score vectors of unique monomers. Then the top 100 principal components (PC) were chosen for clustering. The top 100 PCs can explain around 60% of the total variance (53.6%, 66.3%, and 57.3% for A, G, and T, respectively). We used HDBSCAN [42] for clustering, as it accommodates clusters with different degrees of variation. This helps in monomer subtype detection because the lineage of monomers includes groups that expanded at various ages, leading to differences in expected intra-group variation. HDBSCAN also designates certain outliers as “noise” so we applied nearest neighbor after HDBSCAN to assign a subtype to each data point that was left out by HDBSCAN.

**Fig. 2:**
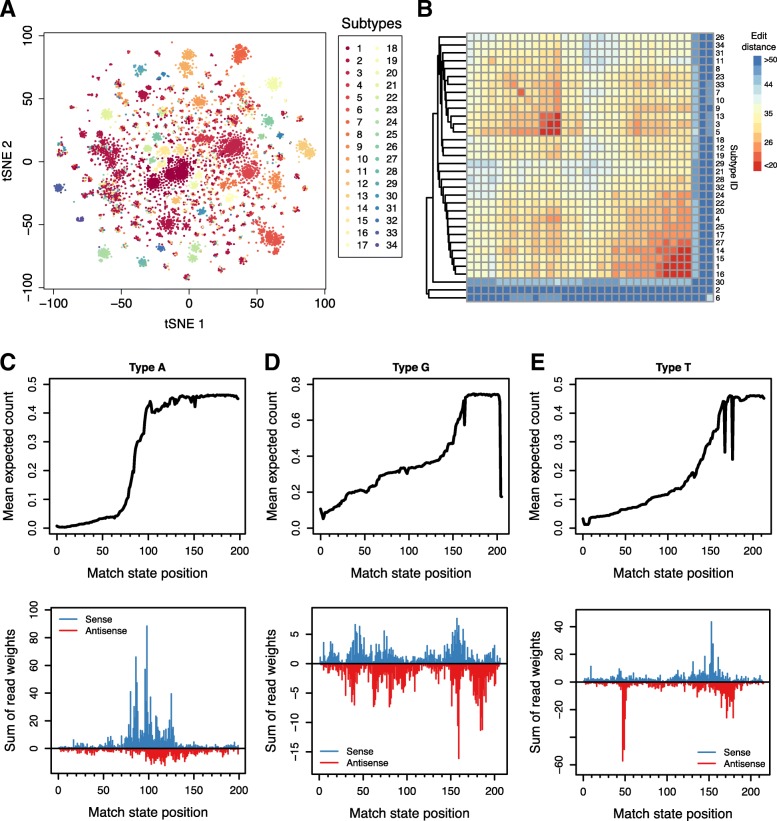
Application of profile-HMM helps identify monomer subtypes and common truncation sites. **a** Visualization by tSNE of subtype identification based on the first 100 principal components using the HDBSCAN algorithm followed by *k*-NN clustering. **b** Heatmap showing sequence variation measured by edit distance for both intra- and inter-group subtypes of the Type A monomers. **c – e** Truncation profile indicated by the posterior expected number of states (top), and weighted read pileup of CAGE-seq data (bottom). Antisense read pileups were visualized as negative values

Using this approach, we identified 34, 6 and 32 subtypes for Type A, G, and T, respectively (Additional file 1: Table S1 – S3). The data used for subtype identification of Type A monomers is shown as a tSNE plot in Fig. 2a. The identified subtypes are numbered based on their counts in a decreasing order. In general, the majority of monomers of each subtype share the same length. We were able to distinguish subtypes that have the same consensus length but differ by nucleotide substitutions. We then measured the divergence of sequence within each subtype and the relationship between subtypes using operation-weighted edit distance, with substitutions and spaces weighted as 1 and 2, respectively. In Fig. 2b, hierarchical clustering for the subtypes of the Type A monomers is shown. Subtype 2, 6 and 30 are outliers, with high variation for both intra- and inter-group comparisons. The consensus lengths and intra-subtype sequence variation of subtypes indicate that our identification method can not only recapitulate the classical A subtype definition based on indels, but also provide further classification to differentiate monomer groups in a finer scale. For example, Subtype 1 corresponds to the majority of the A-I subtype defined by [30]; however, this A-I subtype was also classified to multiple other subtypes such as Subtype 14, 15, etc. We applied the same analysis to G and T subtypes (Additional file 2: Figure S4). Previous studies have reported consensus sequence lengths of 206 bp and 212 bp for Type G and T monomers, respectively [28, 29]. Among the G subtypes we identified, the 206 bp length is the most abundant among all subtypes, while Subtype 3, 4 and 6 have specific deletions (Additional file 1: Table S2). Clustering based on sequence variance shows that Subtype 3 and 4 have low similarity comparing with other subtypes (Additional file 2: Figure S4A). For T subtypes, we found that the length of 214 bp is more abundant than the previously reported 212 bp (Additional file 1: Table S3). Sequence variation analysis indicates Subtype 3 was the only outlier group, with a dominant length of 204 bp, which is closer to that of G subtypes.

### Truncation profiles of monomer types

The truncation site of the 5’-end monomer in a promoter can be considered as the insertion termination site of the corresponding retrotransposon. If the retrotransposon mRNA was complete and fully inserted, this site corresponds to the transcription start site of the parent copy, since retrotransposon promoters must be internal. Previous low-throughput studies reported that many Type A monomers share a common truncation site, while Type G and T often exhibit truncations close to an YY1 binding site [24, 43]. We investigated the truncation profiles of all three monomer types across the mouse reference genome to validate features globally. Using our definition for core monomers (see above), we identified non-core monomers located at the 5’-end of individual L1Md promoters. We excluded any of these monomers if they are located within 20 bp of another retro-element. Then the posterior expected occurrence of each match state was computed for individual monomers (see Methods). We consider the mean of these expected occurrences as the truncation profile of the corresponding monomer type. This approach is more accurate than directly using the length of a truncated monomer to infer the truncation site, because the latter is vulnerable to insertions or deletions within the sequence.

The truncation profiles are shown in Fig. 2 (c – e, top panels). Potential truncation sites are reflected by sharp increases in the curve. Since the x-axis corresponds to the match states in the sense orientation, it can be interpreted as length in basepair of a monomer consensus. Type A monomers show a strong common truncation site indicated by a dramatic increase starting from 70th bp to 100th bp. This indicates the TSS of a Type A monomer is located within the same range. It is similar to a previous estimation of Type A monomer truncation [43], where the authors reported around 1/3 of the total length was commonly truncated. We also observed an intriguing phenomenon around the 100th bp. It is covered by the 7-bp insertion of the sequence ACTCGAG, which has been used to define the A-I subtype, and it also serves as an AP-1 binding site. The analysis of L1 promoter activity done by Severynse, et al. [25] showed that deletion of both AP-1 binding sites can significantly reduce transcription activity of a Type A monomer. However, it is yet unclear whether deleting one of the two binding sites has a similar effect. Since this 7-bp duplication is also included in the Subtype 1 defined in this study (Table S1), it is tempting to speculate that this duplication has greatly increased the activity of Type A promoters. Type G and T truncation profiles support a common truncation near the YY1 binding site (binding motif GCCATCTT), which is located close to the 170th bp in Type G, and the 160th bp in Type T. But the slope is generally smaller than that of Type A, indicating a less fixed TSS (Fig. 2, top panels of d and e). These results indicate that the potential TSS represented by transcription factor binding sites are located very close to the 5’-end termini of individual L1 elements.

To validate the TSS estimation using truncation profiles, we used CAGE-seq data of mouse embryo testes published by the FANTOM5 project [44]. Certain ambiguity of mapping was allowed to effectively capture transcription initiation from highly similar monomer sequences (see Methods). Nevertheless the pileup of reads was weighted to make sure the count of ambiguously mapping reads are correctly normalized. The 5’-end of each mapped read was aligned to a match state in the corresponding monomer profile-HMM via posterior decoding. The weighted read pileup was summed in aggregate for visualization. Here in Fig. 2 (c – e, bottom panels), the time point of E15 is shown, which is close to the stage when DNA methylation is almost completely erased genome-wide during germ cell development. For Type A monomer, there is only strong sense transcription signal, and the highest pileup is located at the 99th bp, overlapping the AP-1 binding site. Type G monomers have less concentrated distribution of read pileup, and the antisense transcription signal (highest at 159th bp) is even stronger than the sense (highest at 158th bp). Type T monomer has a similar bipartite transcription signal, which has a sense peak located at 154th bp and antisense peak at 47th bp. The sense peak of both monomer types are close to the known YY1 binding site. The multimodal distribution of Type G monomer pileup supports its more flat truncation profile curve, which indicates there are potentially multiple transcription start sites. As for the other time points during germ cell development (Additional file 2: Figure S5), the distributions of TSS signals retain a similar shape for all monomer types. The only exception is Type T monomer, which gains a strong TSS at the position around the 10th bp on E16, and this peak persists till E18, suggesting an alternative TSS for the Type T monomers. It is worth noting that due to mapping ambiguity and our strategy of read weighting, this quantification result of transcription initiation cannot be normalized across different time points. We noticed that Type G and T monomers have strong antisense TSS signals, especially for the peak located around the 50th bp of Type T. This indicates that mouse L1MdG and L1MdT elements might also have bidirectional promoters similar to those of human L1HS elements [45, 46].

### Analysis of subtype composition in L1Md promoters

**Fig. 3:**
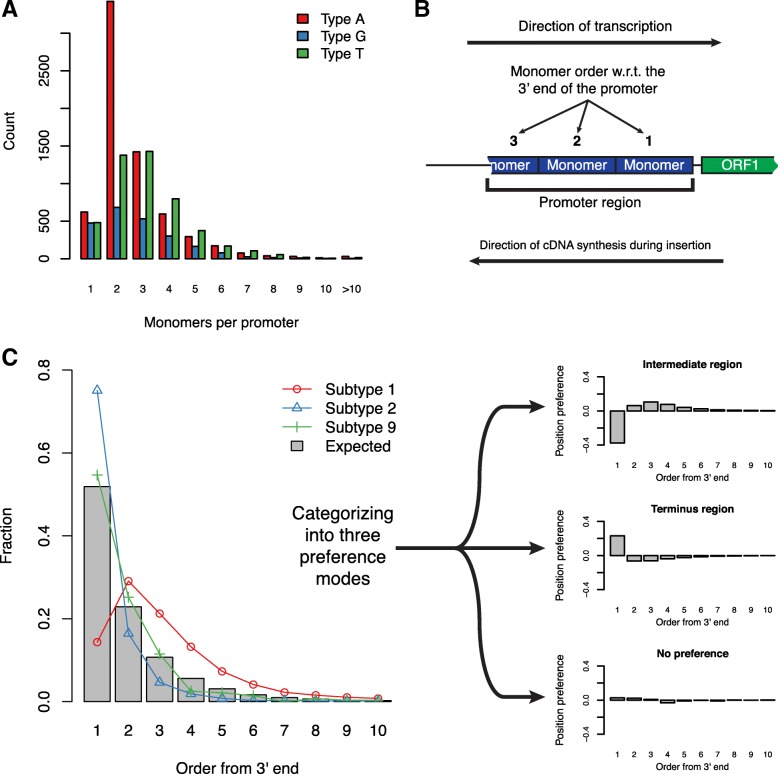
Analysis of monomer subtype composition in L1 promoters shows three position preference modes. **a** Distribution of monomer count per promoter of all three types. Promoters were generated by merging monomers within 20bp of each other. **b** Schematic diagram of ordering monomers based on their placement with respect to the orientation and ORF. **c** Three modes of monomer position preference. The distribution of monomer count for Type A promoters was used (left panel). Three A subtypes were chosen to represent distinct position preference modes, respectively. The three modes are: (1) Subtype 1 corresponding to the intermediate region mode, (2) Subtype 2 corresponding to the terminus region mode, and (3) Subtype 9 corresponding to the no preference mode

We joined proximal monomers as promoter regions, and the types of individual promoters are classified correspondingly to the type of comprising monomers. The statistics of L1Md promoters in the mm10 genome are included in Table 1. It is shown that most of the promoter classification conformed with the classification generated by RepeatMasker based on whole retrotransposons. However, a small number of retrotransposons have mismatched promoter types. The majority of such mismatched types can be attributed to the fact that many L1MdF elements have been identified to possess a Type G or T promoter. The mean counts of monomers per promoter are 2.7, 2.9 and 3.1 for Type A, G and T, respectively. The corresponding distribution is shown in Fig. 3a. The majority of Type A promoters have two monomers, while for the other two types of promoter the distribution is less concentrated and the mode shifts towards 3. This is because Type G and T promoters can have multiple incomplete monomers located close to the 3’-end of the promoter [28]. It is worth mentioning that there are a number of extremely long promoters. The longest promoter is located on chromosome 3, position 6296898 – 6308206 (mm10 reference), which has 50 Type A monomers. However, in the latest version RepeatMasker annotation (using Repeat Library 20140131), it is annotated as part of an L1MdGf_I element, following the subfamily definition introduced by [26].

**Table 1:** Statistics of detected promoters and retrotransposon family classification provided by RepeatMasker (RM)

Retrotransposons annotated by RM		Type A promoters			Type G promoters			Type T promoters		
Family	Count	Count	RM %	p. %	Count	RM %	p. %	Count	RM %	p. %
L1MdA	40,139	6507	16.2	87.3	329	0.8	14.4	184	0.5	3.6
L1MdG	5096	189	3.7	2.5	813	16.0	35.7	248	4.9	4.9
L1MdT	9287	109	1.2	1.5	29	0.3	1.3	3447	37.1	67.4
L1MdF	70,753	453	0.6	6.1	1069	1.5	46.9	1149	1.6	22.5
Others	–	196	–	2.6	39	–	1.7	85	–	1.7

For each detected promoter, its overlap with current annotation of RM was searched. The overlaps were listed in this table with percentage of the corresponding retrotransposons (RM %) and percentage of the corresponding promoter type (p. %). Because not all L1 elements have a promoter, the sum of RM % for each row is not 100

We then investigated whether any specific pattern of subtype composition exists within L1 promoters. In the following analysis of subtype composition within promoters, all monomers including the core ones and truncated ones were used. The subtype assignments for non-core monomers were inferred using the same model trained for the core ones based on PCA and *k*-NN. To consistently label monomers in individual promoters, we ordered the positions based on the direction of the first strand cDNA synthesis during L1 insertion (Fig. 3b), which is opposite to the direction of transcription. Previously it has been observed that certain A subtypes prefer to locate only at position 1, while other subtypes tend to appear in further upstream position [30]. We tested whether our new definition of subtypes for all A, G and T promoters supports this observation. Since 99% of the promoters have no more than 10 monomers, we only considered the positions up to 10. Type A promoters were first analyzed because they do not have truncated internal monomers like Type G and T. We calculated the fraction of individual position orders using all subtypes as the expected distribution. We hypothesized that no subtypes have position preference, then for each subtype the position order should follow this expected distribution (shown in Fig. 3c as the bars; left panel). Then we computed the fractions of position orders for each subtype and compared them with the expected. A position preference value was calculated for each position order by subtracting the background value from the observed one. We summarized three position preference modes from the results of all A subtypes (Additional file 1: Table S4): (1) intermediate region, (2) terminus region and (3) no preference (Fig. 3c, right panel). The list for preference mode of individual A subtypes is shown in Table 2. The first mode is represented by Subtype 1, which has low preference (<−0.1) for the first position and tends to locate in upstream regions from the 3’-end of a monomer array. This kind of subtype contributes the most to promoter extension. The second mode, for example Subtype 2, favors location of the 3’-end terminus (≥0.1 preference for position order of 1). The third mode shows no preference value beyond the range of [−0.1,0.1) at any position, such as Subtype 9. The only un-categorized subtype after these criteria is Subtype 30, which has a position preference value greater than 0.1 at the position order of 2 (Additional file 1: Table S4). Subtypes of G and T monomers also follow these three modes (Additional file 1: Table S5, 6). However, we had to extend the definition of terminus region mode for these two types to the first 3 position orders to accommodate the truncated monomers. Therefore for G and T subtypes, we changed the condition for the terminus region mode to greater than 0.1 preference value in any of the first three position orders. The preference mode summary of G and T subtypes can be found in Additional file 1: Table S7.

**Table 2:** List of subtypes of three position preference modes of A subtypes. Subtype 30 has an outlier mode and thus it does not belong to any of these three modes

Preference mode	Subtype list	Total count
Intermediate region	1, 14, 15, 16, 17, 20, 22, 23, 24, 25, 27, 28, 32, 33	4106
Terminus region	2, 3, 5, 6, 7, 10, 11, 13, 31	4984
No preference	4, 8, 9, 12, 18, 19, 21, 26, 29, 34	2108

Combining the position preference modes of individual subtypes and their sequence divergence shown in Fig. 2b, we found that there is particular relationship between subtypes with similar sequence and their position preference. Excluding the special case of Subtype 30, the clustering result based on sequence also separates most subtypes by their position preference mode, especially for the subtypes with the intermediate region mode (Additional file 2: Figure S6A). For G and T subtypes, this pattern still applies (Additional file 2: Figure S6B and C). We speculate that this phenomenon is due to the expansion pattern of L1Md promoters during evolution. Our results indicate that the mechanism of monomer array extension functions through inserting monomers to the upstream of current L1Md promoters, and it uses monomers located in the intermediate region as templates, rather than those located in the terminus region. Thus subtypes observed to have position preference at terminus regions are not likely to contribute to promoter extension in this mechanism. On the other hand, the subtypes showing no preference in positioning distribute uniformly along the positions of L1Md promoters. This suggests an alternative extension mechanism may exist, capable of using all monomers within the promoter as insertion template.

### Functional annotation of L1Md promoters

The set of retrotransposition-competent L1 elements has been well defined in L1Base2 [27], in which the competence is characterized based on both promoter and ORF completeness. Since L1Base2 uses the L1Md classification generated by RepeatMasker, we are interested in investigating the transcription potential for activity of individual L1Md promoters based on our classification of monomer subtypes. Because of the high copy number of L1Md elements in the genome, it is not very effective to quantitatively estimate the transcription levels of L1Md elements through typical RNA-seq data analysis. Therefore to evaluate the capability of initiating transcription of individual L1Md promoters, we chose to use promoter DNA methylation as the measurement to indirectly estimate the activity of L1Md promoters. As mentioned before, the piRNA regulation pathway is responsible for repressing actively transcribed retrotransposons through piRNA-guided *de nove* DNA methylation in developing germ cells. We chose public datasets of certain knockout (KO) experiments related with the piRNA regulation pathway for identification of active L1Md promoters, by comparing with the methylation levels in paired wild type (WT) samples included in their corresponding studies [23, 47, 48]. This is because during germ cell development, most of the active L1Md promoters are suppressed by deposition of DNA methylation, and therefore they cannot be directly distinguished from other inert promoters that are routinely methylated together with most intergenic regions. With methylation data after knocking out key factors of the piRNA regulation pathway, active L1Md promoters can be identified by measuring the methylation loss. The Dnmt3L KO data generated by [48] was used as a negative control, because this enzyme is known to be responsible for methylating retrotransposon regions during male germ cell development [16, 49, 50]. The other factors, Mili, Miwi2 and Pld6, represent piRNA regulation at different stages of spermatogenesis [51, 52].

**Fig. 4:**
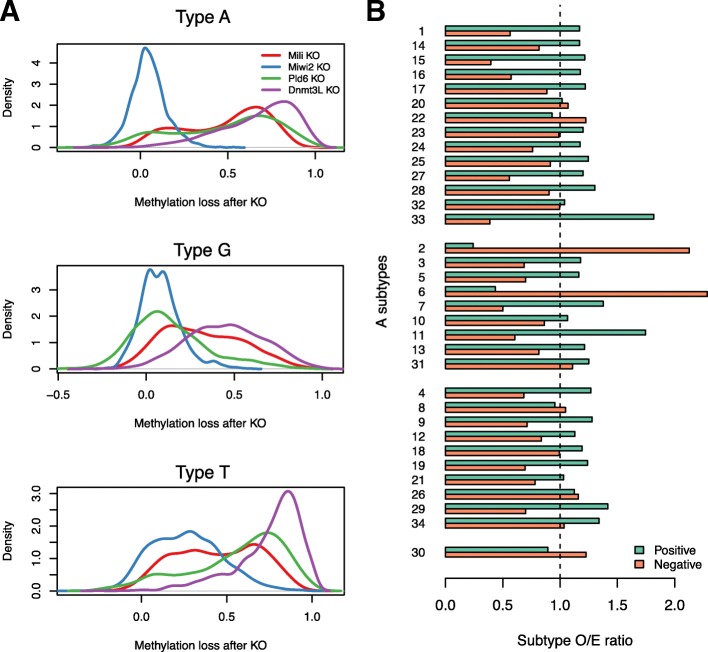
Differential response after piRNA pathway related KO experiments implies distinct L1Md promoter activity. **a** Distribution of methylation loss after KO experiments. **b** O/E ratios of A subtypes grouped by methylation loss after the Mili KO experiment. The groups were defined by the extent of methylation loss, including Positive (loss ≥0.5) and Negative (loss <0.5). The subtypes were arranged based on their position preference modes, which are intermediate region (top), terminus region (middle), and no preference (bottom), while Subtype 30 is listed as the outlier group

For each L1Md promoter, we calculated its average methylation level in all KO experiments and WT samples. A minimum coverage filter was applied to obtain precise estimation of methylation levels (see Methods). After filtering, there are in total 4187, 1359 and 2411 promoters of Type A, G and T, respectively. The distributions of the methylation loss can be seen in Fig. 4a, grouped by promoter type. The negative control Dnmt3L KO showed expected methylation loss, despite Type G promoters losing less methylation. Since most of the promoters were hypermethylated in WT samples, the observed low methylation loss after KO experiments could not have been caused by hypomethylation in WT (Additional file 2: Figure S7). All three types exhibit diverged responses in the Mili KO comparison. However, following Miwi2 KO only the Type T promoters have methylation loss. For the KO of Pld6, the majority of Type A and T promoters were demethylated, while most of the Type G promoters retained their methylation.

We then investigated the diverged response after Mili KO. All promoters were separated to two groups, labeled as positive and negative of methylation loss, based on the threshold at 0.5 methylation difference. To explore whether there are any subtypes highly correlated with positive or negative response, we calculated an observed to expected (O/E) ratio for each of the subtypes in both groups. The expected distribution assumes all subtypes are randomly distributed across all promoters, and the fractions of individual subtypes should be proportional to the counts of subtypes in all promoters passing the coverage threshold. The O/E ratios of Type A subtypes are shown in Fig. 4b. The most extreme values belong to Subtype 2 and 6 in the “negative loss” group. We showed (above) that these two subtypes are mostly positioned at the 3’-end of promoters, and their sequences diverged from other subtypes. This suggests that these two subtypes have already lost the function as a promoter, and if one L1Md promoter only has these two subtypes, it is very likely that such a promoter is inert during germ cell development. In general, subtypes that have a preference in intermediate regions are more enriched in the positive group, while terminus region subtypes show the opposite trend. We observed a similar pattern for G and T subtypes after Mili KO (Additional file 2: Figure S8, A and B). For the Miwi2 KO, we used a lower cut-off for positive methylation loss (loss ≥0.3), given that the response after KO is not as strong as the other factors. The result of enrichment analysis indicates that the regulation targets of Miwi2 on Type T promoters are similar to those of Mili (Additional file 2: Figure S8C). Because Mili and Miwi2 are active in different stages of spermatogenesis, it is yet unclear whether the overall negative response for Type A and G promoters after Miwi2 KO is due to the limit of available time points of data used in this analysis. It is intriguing that even Type G and T promoters have divergent responses after Miwi2 KO, despite the high sequence similarity between Type G and T monomers. On the other hand, since Mili binds to primary piRNA and Miwi2 binds to secondary piRNA, the differential response after Miwi2 KO might be associated with the increased antisense transcription from Type T promoters shown in Fig. 2e.

## Discussion

In LINE-1 elements of rat (name as L1Rn families following the species name *Rattus norvegicus*), tandem repeats of length around 600-bp were discovered in promoter regions [53]. However, when we applied our pipeline for the rat genome assembly (rn6), we could not detect a monomer with length close to 200 bp as in the *Mus* species. We made two attempts, one with the same detection setup starting from a mouse monomer consensus sequence search by nhmmer, and the other one directly starting from TRF detection in 5’ UTR located by homologous search of L1 ORF1 sequence. Although the first attempt might be limited by low sequence similarity between potential rat monomers and the ones in mouse, the second should have been able to discover tandem repeat structure if there is any in the 5’ UTR of L1Rn retrotransposons. We did observe a small number of mouse-monomer-like sequences in the rat genome, which belong to retro-elements of the Lx family. Because the Lx family is common in rodents [54], this result suggests that monomers around 200 bp may have existed earlier in Rodentia, but proliferated specifically within *Mus*. We observed the rat-specific 600-bp tandem repeats in only few L1Rn elements, with copy number no more than 2 per promoter. Taken together, these observations suggest monomers around 200 bp have undergone a coupled proliferation and within-element extension in *Mus* species, and this interaction has greatly enhanced the potential for activity of L1Md elements.

We chose to exclude the F type monomer from our analysis because it is the ancestral sequence of the G_f_ and T_f_ types, and it shares high sequence similarity with these two types. Our current implementation of the detection pipeline does not handle hierarchy; therefore monomers which were found to have ambiguous classification would require manual distinction. However, in practice we found that the detection results of nhmmer for G_f_ and T_f_ monomers have almost no overlap. So in the current scope including only A, G_f_ and T_f_ monomers, we did not need to deal with issues of ambiguity in classifying monomers. We also demonstrated that this pipeline can be applied to other starting regions instead of nhmmer detection results for potential monomers, as mentioned before in the monomer investigation for the rat genome.

The number of monomer-containing L1Md promoters does not necessarily equate to the number of full-length L1Md elements, because there are a number of disruptions which can separate a promoter from its ORFs. For example, one full-length L1 element can be interrupted (rendered discontiguous) by another retrotransposon insertion, and some families of L1, such as L1MdV and L1MdN, have monomer-free promoters [26, 55]. It is also known that during L1 insertion, an inverted 5’ UTR could be created next to its downstream part [56]; we observed this phenomenon in our analysis (data not shown). Because the inversion information is not included in current retrotransposon annotation, such promoters will be considered as independent retrotransposons, thus omitted by full-length statistics. We found that the number of promoters detected by our pipeline exceeds the number of full-length L1 elements reported by [26], suggesting the possibility that an active L1 promoter might drive transcription of arbitrary downstream DNA unless effectively suppressed. The classification of an L1Md element does not always suggest its promoter type, as shown in Table 1. This is because the typical classification of retrotransposon is not only based on 5’ UTR, but also ORFs and 3’ UTR. Given the frequent recombination between retrotransposons, it is possible to observe an L1 element with a mismatching type of promoter [33].

The mixture of position preference modes implies that more than one promoter extension mechanism exists. Previously two potential models were proposed: expansion during cDNA second strand priming and duplication through unequal crossing-over [30, 57]. The latter mechanism certainly exists, but lacks any quantitative support; the requirements for the former mechanism are much less clear. In our analysis of monomer composition, we have seen evidence supporting both models. For example, the abundant Subtype 1 of the A monomers follows the intermediate position preference mode. This supports the model that additional monomers can be inserted into the 5’-end of the promoter, resulting a “stepwise” expansion, as introduced by [30]. However, it is unlikely under this model to observe extremely long promoters, such as the example we mentioned in the “Results” section, where a promoter includes 50 monomers. This array of A monomers is attached to an L1MdG element, and its subtype composition is not homogeneous. This extremely long promoter is likely a consequence of unequal crossing-over, rather than sequential insertions of individual monomers [30].

The enrichment pattern of subtypes after KO experiments implies that certain subtypes of monomers have lost their potential as promoters. Unfortunately, we yet still could not distinguish the active monomers within a monomer array. This is due to the high sequence similarity between individual monomers, even if they belong to different subtypes. The difference between subtypes is currently beyond the resolution of data we have analyzed, and thus it is very difficult to attribute methylation changes to specific monomers. The number of monomers in a promoter also plays an important role. For example, for long promoters which were found to lose methylation after KO, it is possible that they contain inert subtypes as well as active ones. But due to the limit mentioned before, they are not distinguishable and thus the power of enrichment analysis could be reduced.

## Conclusions

We developed a computational pipeline which is capable of detecting monomers as the L1Md promoters. Application of this pipeline in multiple mouse species discovered monomers successfully. Comprehensive comparison of L1Md promoters between species is not yet possible due to incomplete genome assemblies, but we were able to verify the existence of an important monomer subtype in all species using our profile-HMM. The discovery of the 7-bp insertion related to Type A monomer in the sequencing data of all analyzed *Mus* species revealed that this important insertion has emerged prior to the diversification of *Mus*. The classification of monomer subtypes in the mm10 genome based on genome-wide data reveals detailed grouping of all the three types of monomers. The previous observation of positioning pattern for specific subtypes is verified by our study, and we further supplemented it by summarizing the three modes of position preference. We showed that this preference mode is related to the sequences of individual monomers, and it indicates that the expansion of L1Md promoters is a combined effect of both a monomer being duplicated and crossing over of promoter fragments. Analysis of methylation change after KO of certain piRNA pathway factors suggests divergent response of L1Md promoters, with distinct modes of interaction with the piRNA pathway. Overall, the promoter-centric annotations generated through this study provide a useful resource for annotation and functional studies of retrotransposon promoters.

## Methods

### Iterative process of profile-HMM construction

The first step of model construction is to learn the model length of profile-HMM. The consensus sequences of each monomer type, A, G and T are used to generate a starting profile-HMM using the hmm-build tool in HMMER [58]. Then nhmmer is used to scan the genome with options “–max –cut_ga” for maximum sensitivity. The search result is filtered with minimum bit score of 60 to remove potential false positives. Each detected monomer is extended by 20 bp on both sides such that they can be collapsed to form promoter regions even when small gaps exist. Then TandemRepeatsFinder [37] (TRF) is used on the reverse-complement sequences of those potential promoters to detect the repeating pattern, in order to avoid the effect of the common 5’ truncation. The parameters of TRF are set to “2 5 5 80 10 50 500 -h -ngs”, which restricts the maximum length of repeating pattern to 500 bp. All patterns that have more than two copy numbers in a promoter and length within range from 180 bp to 220 bp are kept. Then 500 of such patterns are randomly sampled and restored to their original orientation to construct a multiple sequence alignment using MUSCLE [59]. A initial profile-HMM is built from this multiple alignment with the model length estimated through the MAP algorithm introduced by [36]. The MAP algorithm is tuned such that more columns would be included in the profile-HMM to retain as much information as possible in the resulting model. Posterior decoding is then applied to all promoter sequences using this initial profile-HMM for an initial detection of monomers. The optimal model length is set to the median of the top 8 most common lengths of the detected monomers.

The next step is to iteratively refine the emission parameters of the initial profile-HMM. In each iteration, posterior decoding is applied for all promoter sequences with 500 bp flanking regions for better separation of the foreground and background sequences. Among the detected monomers that are no more than 20 bp longer or shorter than the optimal model length, 500 are sampled from the top 33% scoring ones to construct a multiple sequence alignment. A reference sequence is included in this multiple alignment to guide model construction, such that the model length stays unchanged. This alignment is then used for training the emission parameters to get an updated profile-HMM. The iteration process is terminated when the Jaccard index of the regions identified as monomers between two iterations becomes higher than 0.95.

### Monomer subtype identification

Fisher scores of the core monomers are centered for each dimension. Then PCA is performed using the prcomp function in R. The first 100 PCs are chosen for HDBSCAN using the dbscan package, with MP (min points) set to 20 for Type A and T; MP is set to 10 for Type G subtype identification. Core monomers that are not classified by HDBSCAN, as well as other non-core monomers, are later assigned to an existing subtype based on nearest neighbor clustering with k set to 3.

### Abundance estimation using genomic-sequencing data

To quantitatively estimate the abundance of L1 elements and L1Md promoters in a genome, nhmmer is used to search for the consensus sequences among genomic sequencing reads. The consensus sequences include the sequence of L1 _spa_ ORF2 (GenBank accession AF016099.1), and the consensus sequence of Type A monomer reported in Additional file 2: Figure S2. A minimum bit score of 20 before bias correction is used for filtering out noise. Reads that are detected to be related with Type A monomer are then used for detection of the 7-bp insertion. The expected occurrences of match states are calculated using the following equation.

Given a profile-HMM with parameters ***θ*** and the input sequence ***X***, we denote the forward probability as *α*_*k*_(*i*), and the backward probability as *β*_*k*_(*i*), where *k* represents the hidden state, and *i* is the position of the input sequence. Then by the definition of forward and backward probabilities, the posterior expectation for the occurrence of state *k* in the sequence is:

$$\begin{array}{*{20}l} \mathbb{E}(k|X, \boldsymbol{\theta}) & = \sum_{i=1}^{N} \Pr(y_{i} = k|X, \boldsymbol{\theta})\\ & = \sum_{i=1}^{N} \frac{\alpha_{k}(i)\beta_{k}(i)}{\Pr(X|\theta)}. \end{array} $$

If no overrepresentation exists for any match state, this value is usually close to 1. Based on the profile-HMM we trained for Type A monomers, reads that have over 1.2 of posterior occurrence values of match states 98 – 104 can be considered to carry the 7-bp insertion.

### CAGE-seq analysis and alignment to profile-HMM states

Raw CAGE-seq data chosen from the FANTOM5 project [44] were processed by cutadapt [60] for adaptor removal. All reads were mapped using bowtie-1.0.0 [61] with end-to-end mode allowing at most 3 mismatches per alignment and “–best”. Mapping ambiguity was set to allowing maximum 100k mappable locations by the “-m” option. Then for every reported alignment of each individual read, its weight was set to the fraction of total mapped locations, such that the sum of all locations of one read is 1.

For each mapped read, its 5’-end position was used for aligning to a match state of the monomer profile-HMM. This was done by computing posterior decoding of the entire promoter sequence, and then the match state located at the mapping location was used. Alignments to an insertion state were discarded.

### Methylation analysis of L1Md promoters

Processed public methylation data were obtained from MethBase [62]. For each promoter region, we required it to cover at least 3 CpG sites with mapped reads and 10 observations of methylation. This observation is defined by one read covering a CpG site. Then the average methylation level was computed by the weighted average of methylation at individual CpG sites.

## Additional files

Additional file 1 **Table S1**: Subtypes of A monomers in the mm10 genome. **Table S2**: Subtypes of G monomers in the mm10 genome. **Table S3**: Subtypes of T monomers in the mm10 genome. **Table S4**: Position preference values of A subtypes. **Table S5**: Position preference values of G subtypes. **Table S6**: Position preference values of T subtypes. **Table S7**: Position preference modes for G and T monomer subtypes. **Table S8**: Statistics of reads related with L1Md in various species. (XLSX 58 kb)

Additional file 2 **Figure S1**: Topology of the profile-HMM implemented for monomer detection. **Figure S2**: Distribution of unassembled nucleotides with respect to detected Type A monomers. **Figure S3**: Consensus sequences extracted from profile-HMM trained for mm10 monomers. **Figure S4**: Heatmap of intra- and inter-subtype edit distances. **Figure S5**: Weighted read pileup of CAGE-seq data at various time points during germ cell development. **Figure S6**: Hierarchical clustering of subtypes based on edit distance. **Figure S7**: Scatter plot of L1Md promoter methylation before and after different KO experiments. **Figure S8**: O/E ratios of G and T subtypes after KO experiments. (PDF 3395 kb)

## Abbreviations

HMM: Hidden markov model
KO: Knock-out
NN: Nearest neighbor
ORF: Open reading frame
PCA: Principal component analysis
piRNA: Piwi-interacting RNA
TRF: Tandem repeats finder
TSS: Transcriptions start site
UTR: Untranslated region
WT: Wild type
