# Supplementary material for: Subtype classification and functional annotation of L1Md retrotransposon promoters
Source: Mob DNA. 2019 Apr 8;10:14. doi: 10.1186/s13100-019-0156-5 (PMC6454616; doi:10.1186/s13100-019-0156-5)
Supplement: Supplementary file 2 — Figure S1: Topology of the profile-HMM implemented for monomer detection. Figure S2: Distribution of unassembled nucleotides with respect to detected Type A monomers. Figure S3: Consensus sequences extracted from profile-HMM trained for mm10 monomers. Figure S4: Heatmap of intra- and inter-subtype edit distances. Figure S5: Weighted read pileup of CAGE-seq data at various time points during germ cell development. Figure S6: Hierarchical clustering of subtypes based on edit distance. Figure S7: Scatter plot of L1Md promoter methylation before and after different KO experiments. Figure S8: O/E ratios of G and T subtypes after KO experiments. (PDF 3395 kb) [file 13100_2019_156_MOESM2_ESM.pdf]

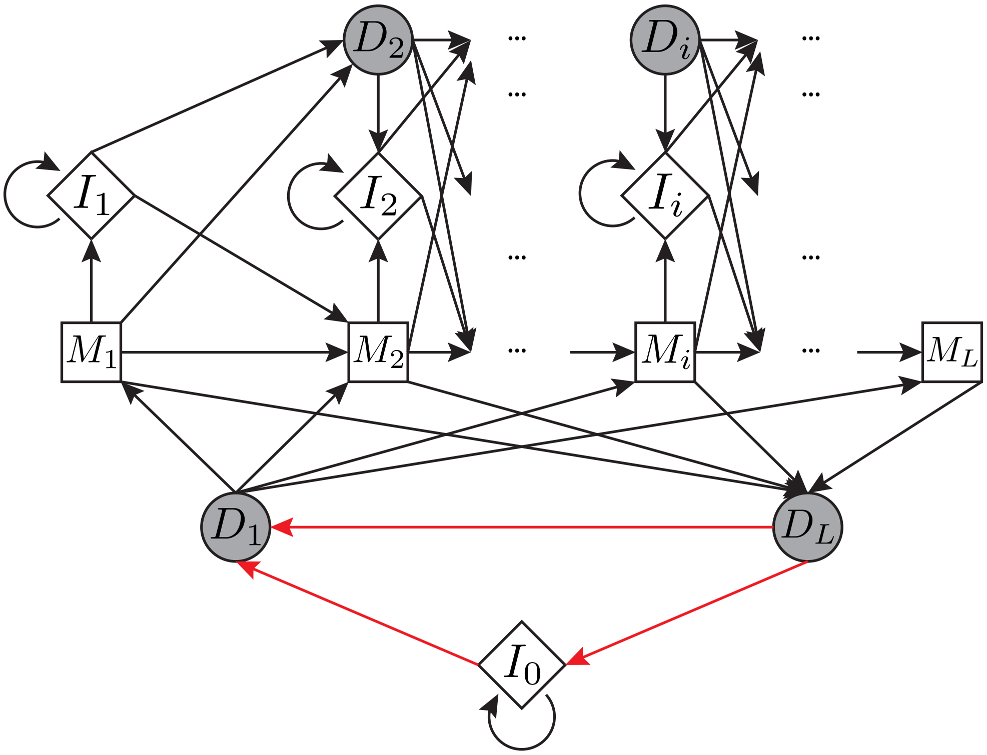

Figure 1: Topology of the profile-HMM implemented for monomer detection. It features with a transition from the last deletion state D_L to the first deletion state D_1. This transition enables looping occurrences without non-homology nucleotide (represented by the state I_0). The transition edges which are customized for this study are highlighted in red.

A

```
       1                                                  51
A_mm10 GCGCCTGCCCCAATCCAATCGCGCGGAACTTGAGACTGCGGTACATAGGG AAGCAGGCTACCCGGGCCTGATCTGGGGCACAAGTCCCTTCCGCTC----
A-I    GTGCCTGCCCCAATCCAATCGCGCGGAACTCGAGACTGCGGTACATAGGG AAGCAGGCTACCCGGGCCTGATCTGGGGCACAAGTCCCTTCCGCTCGACT
       * **************************** ******************* **********************************************

       101                                                151
A_mm10 ---CACTCGAGCCCCGGGCTACCTTGCCAGCAGAGTC--GCCCGACACCC GCAAGGGCCCACACAGGACTCCCCACGGGATCCTAAGACCTCTGGTGAGT
A-I    CGAGACTCGAGCCCCGGGCTACCTTGCCAGCAGAGTCTTGCCCAACACCC GCAAGGGTCCACACGGGACTCCCCACGGGACCCTAAGACCTCTGGTGAGT
           *********************************  **** ****** ******* ****** *************** *******************

       201
A_mm10 GGAACACA
A-I    GGATCACA
       *** ****
```

B

```
       1                                                  51
G_mm10 TGGTGAGAGCACAGGGGTCTGCCCGGCCTGAGAGGTTTGTGCCTCAGGCG CCGGCGGGAGCCTTCTTGGCTCCGGGACTCCGCGGAGGGCAGGCTGCACG
G      ---TGAGAGCACGGGGGTCTGCCNGGCCTGAGAGGTTTGTGGCACAGGCG CCGGCGGGAGCCTTCTTGGCTCCGGGACTCCGCGGAGGGCAGGCTGCACG
          ********* ********** ***************** * ****** **************************************************

       101                                                151
G_mm10 GGTGACCGTGTGGAATACAGAG-GCCAGCCGTTTCTGGGACGGGCGAGAG CCACAGAG--CTTCTGAGGCGGCGCCATCTTCGGCTCCAGACAACCGGCC
G      GGTGACCGTGTGGAATACAGAGTGCCAGCCGTTTCTGGGACGGGCGAGAG NNNCAGAGNNCTTCTG-GGCGGCGCCATCTTCAGCTCCAGACAGCCGGCC
       ********************** ***************************    *****  ****** *************** ********** ******

       201
G_mm10 ACCTTCCTG-
G      ACCTTCCTGG
       *********
```

C

```
       1                                                  51
T_mm10 GGTGCGCCAGAGAACCTGACAGCTTCTGGAACAGGCAGAAGCACAGAGGC GCTGAGGCAGCACCCTGTGTGGGCCGGGGACAGCCGGCCACCTTCCGGAC
T      -------------------------------------------------- -------------------------------------------------C
                                                                                                          *

       101                                                151
T_mm10 CAGAGGACAGGTGCCCGCCCGGCTGGGGAGGCGGCCTAAGCCACAGCAGC AGCGGTCGCCATCTTGGTTCCGGGACTCCGCCGAACTTAGGAAATTAGTC
T      CAGAGGACAGGTGCCCGCCCGGCTGGGGAGGCGGCCTAAGCCACAGCAGC AGCGGTCGCCATCTTGG-TCCGGGAC-CCGCCGAACTTAGGAAATTAGTC
       ************************************************** ***************** ******** ***********************

       201                                                251
T_mm10 TGAACAGGTGAGAG------------------------------------ --------------------------------------------------
T      TGAACAGGTGAGAGGGTGCGCCAGAGAACCTGACAGCTTCTGGAACAGGC GGAAGCACAGAGGCGCTGAGGCAGCACCCTGTGTGGGCCGGGGACAGCCG
       **************

       301
T_mm10 -------------
T      GCCACCTTCCGGA
```

Figure 2: Consensus sequences extracted from profile-HMM trained for mm10 monomers. **A**: Type A monomer consensus sequence. The consensus of the 208 bp long A-I subtype defined by Schichman, et al. was used for comparison. **B**: Type G monomer consensus sequence. The G_f monomer consensus reported by Goodier, et al. was used for comparison. **C**: Type T monomer consensus sequence. The T_f monomer consensus reported by Goodier, et al. was used for comparison.

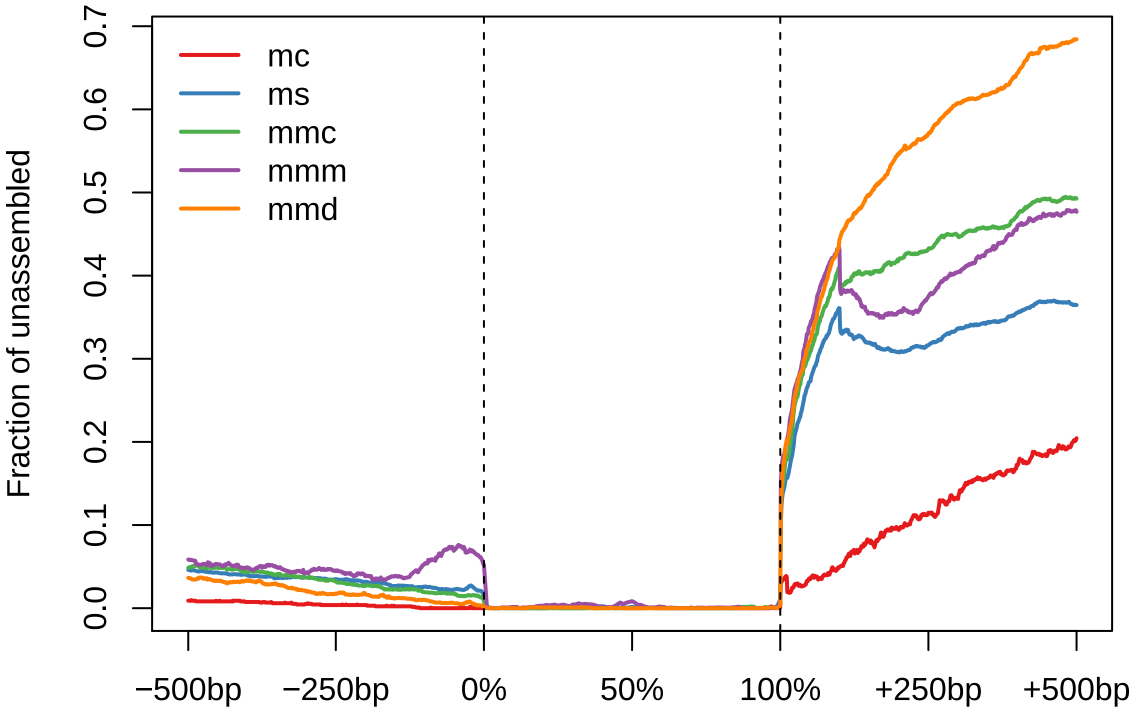

Figure 3: Distribution of unassembled nucleotides with respect to detected Type A monomers. In the x-axis, detected monomers were joined to form potential promoter regions. Then all promoter regions were oriented and scaled to align for the center region of relative percentage. Flanking regions include both up- and downstream 500bp. For each position, the fraction of unassembled nucleotides was calculated and plotted.

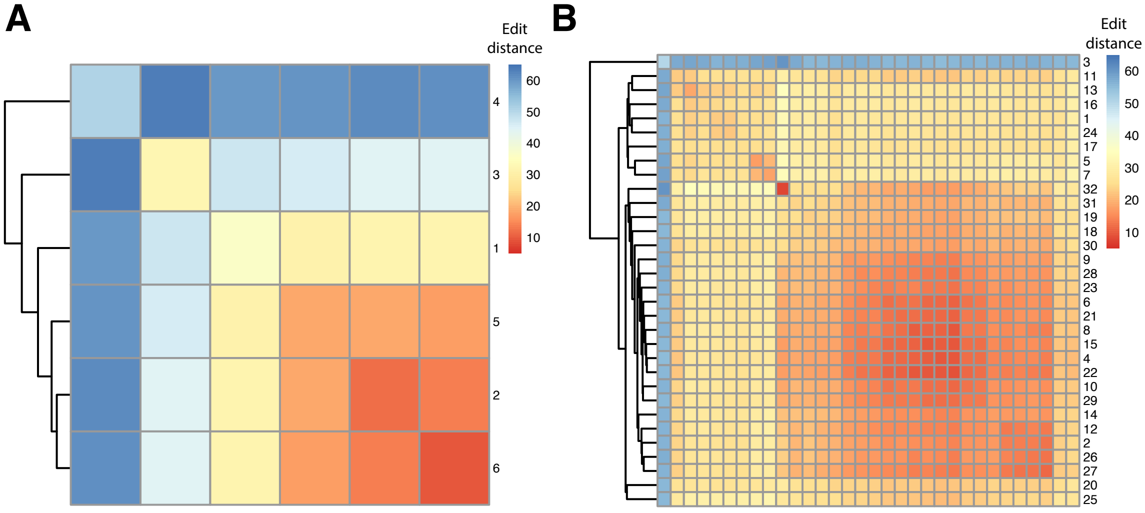

Figure 4: Heatmap of intra- and inter-subtype edit distances. **A**: Identified G subtypes. **B**: Identified T subtypes.

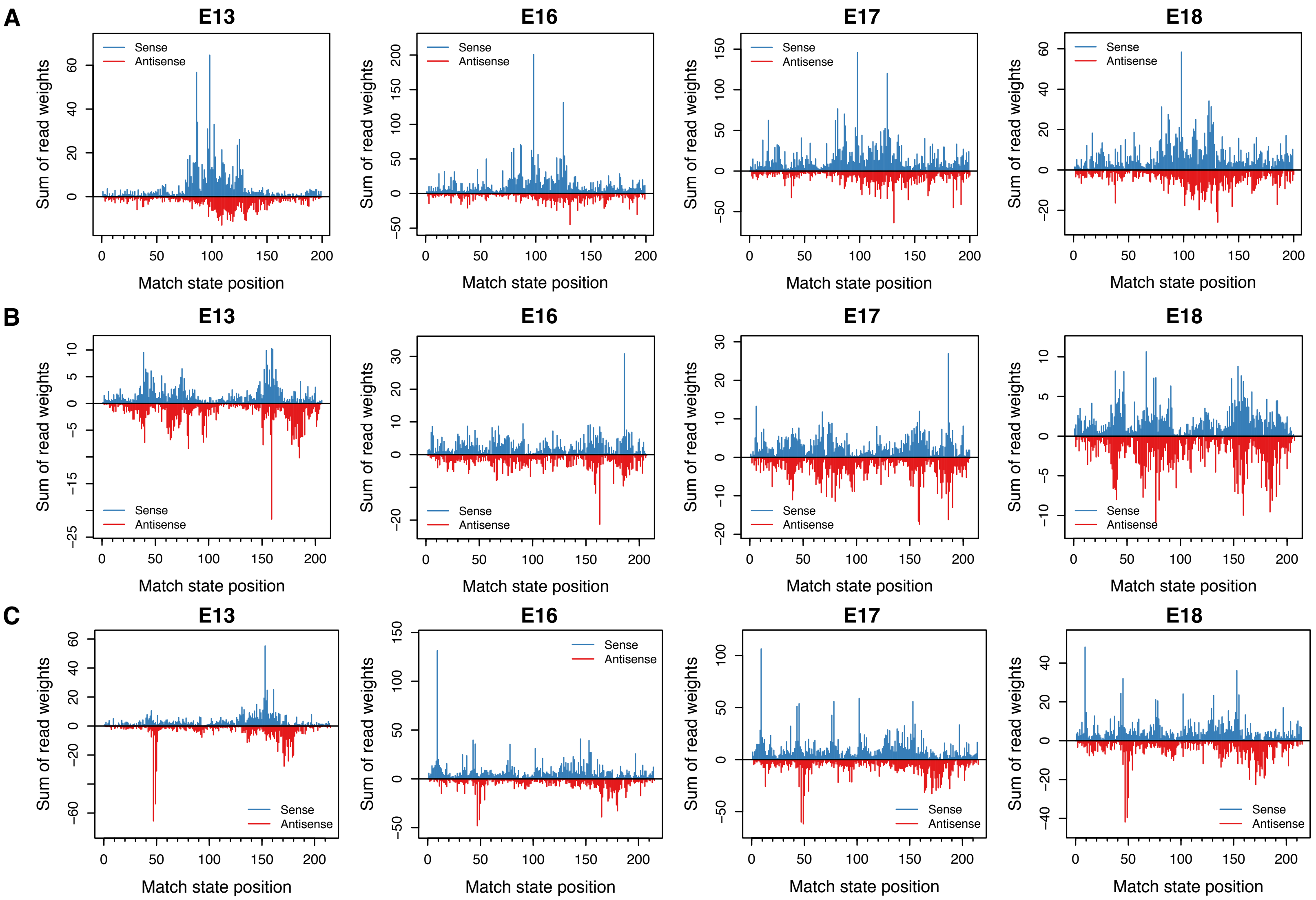

Figure 5: Weighted read pileup of CAGE-seq data at various time points during germ cell development. **A**: Pileup of Type A monomers. **B**: Pileup of Type G monomers. **C**: Pileup of Type T monomers.

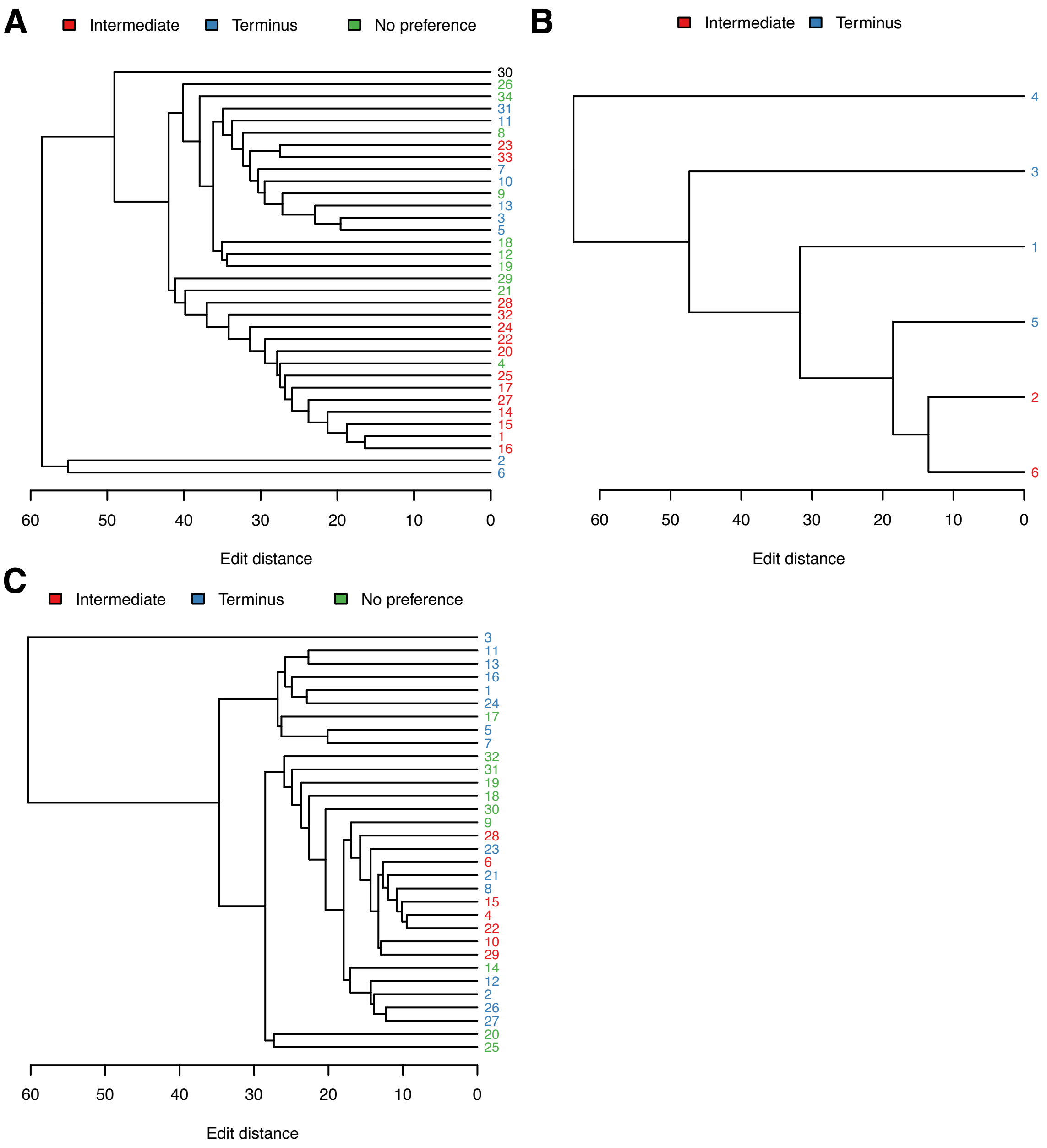

Figure 6: Hierarchical clustering of subtypes based on edit distance. The subtypes are labeled based on their position preference mode. **A**: Subtypes of A monomers. **B**: Subtypes of G monomers. **C**: Subtypes of T monomers.

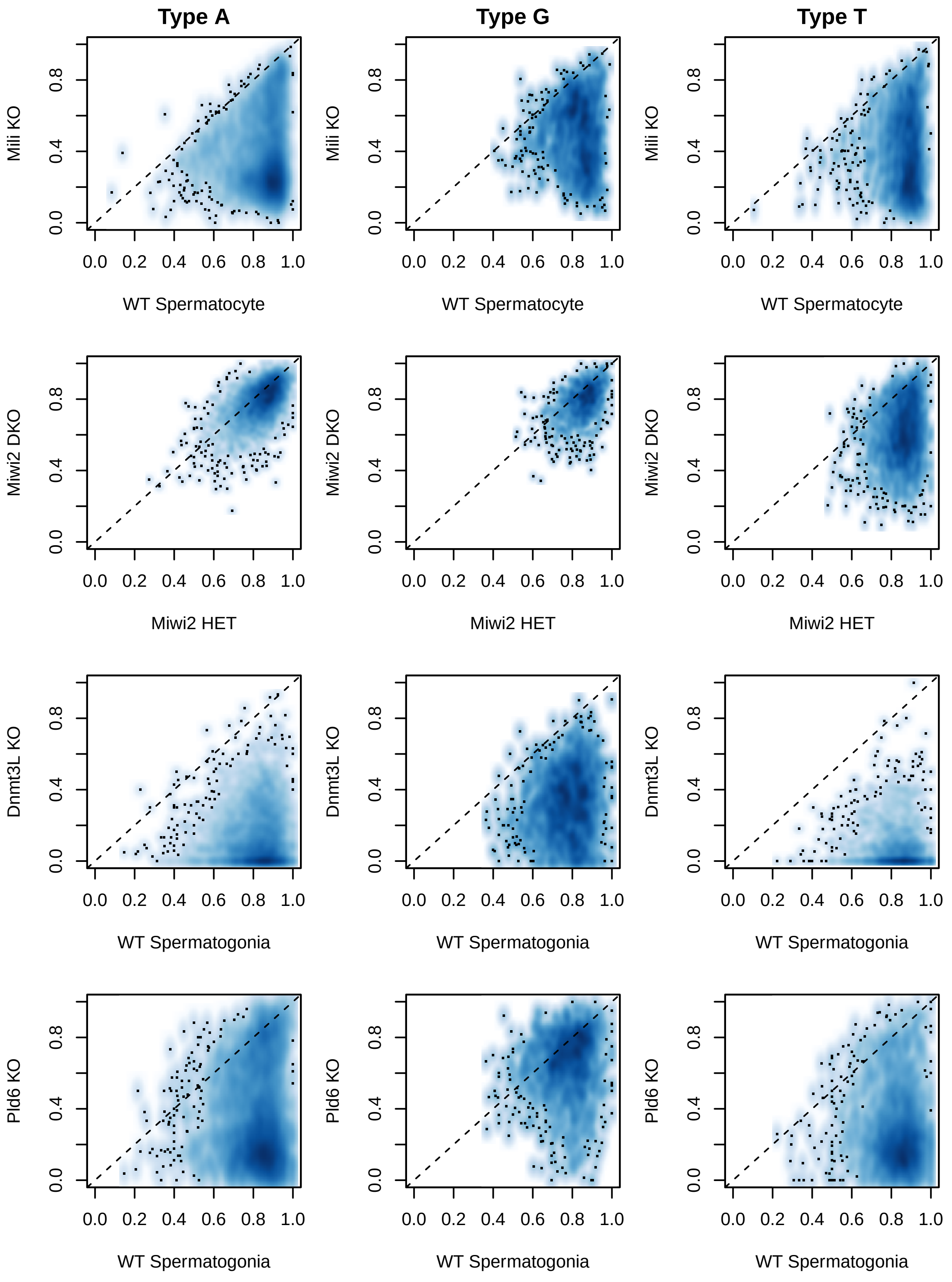

Figure 7: Scatter plot of L1Md promoter methylation before and after different KO experiments. In the comparison of Miwi2, HET stands for the heterozygous genotype Miwi2$^{+/-}$, and DKO stands for double knockout, Miwi2$^{-/-}$.

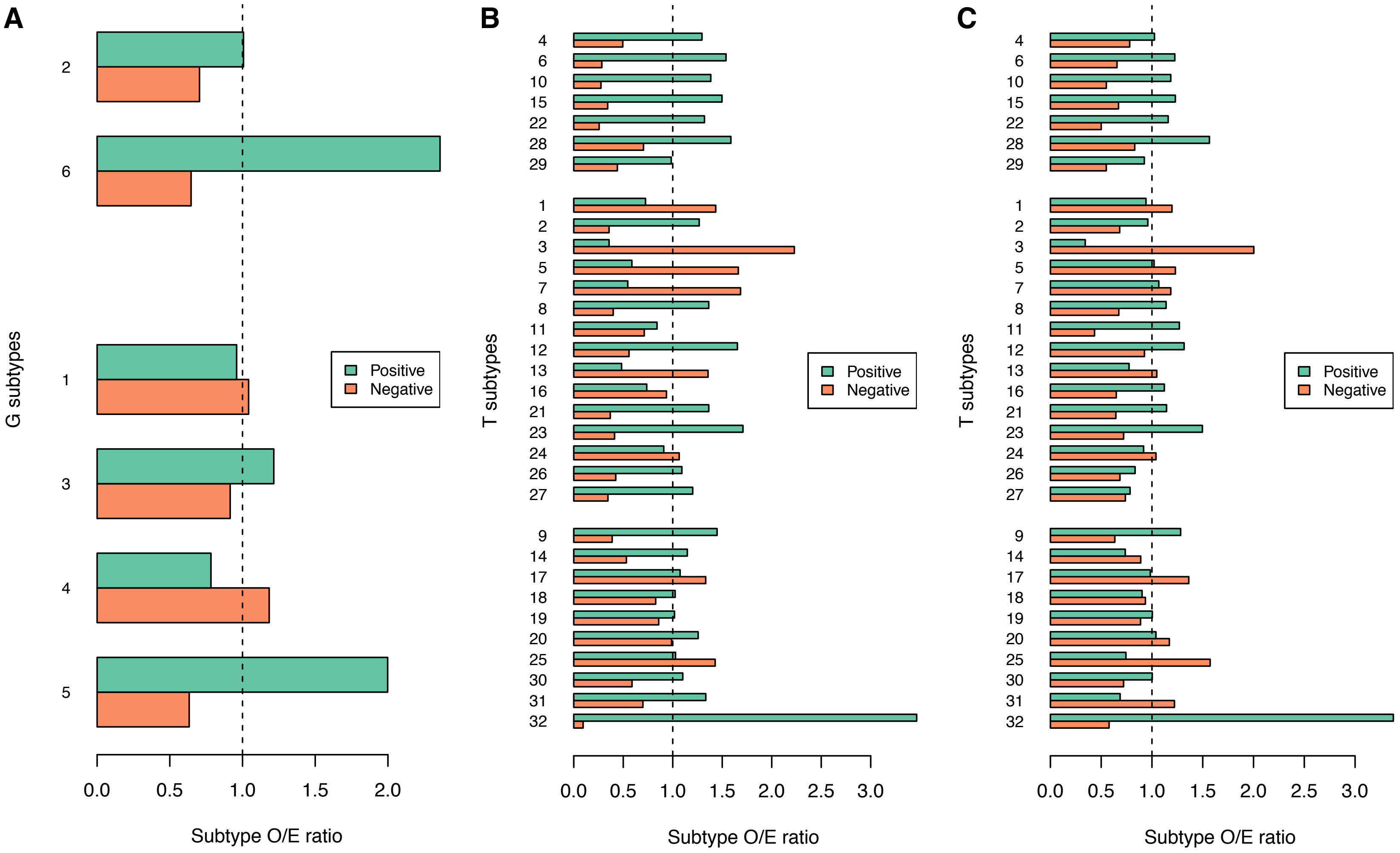

Figure 8: O/E ratios of G and T subtypes after KO experiments. **A**: O/E ratios of G subtypes after Mili KO are grouped by intermediate region (top) and terminus region (bottom). **B**: O/E ratios of T subtypes after Mili KO are grouped by intermediate region (top), linker region (middle), and no preference (bottom). **C**: O/E ratios of T subtypes after Miwi2 DKO are grouped by intermediate region (top), linker region (middle), and no preference (bottom). Note the threshold for defining positive and negative for the Miwi2 DKO is 0.3 instead of 0.5.
